# Transcription coactivator and lncRNA duet evoke Hox genes

**DOI:** 10.1371/journal.pgen.1006797

**Published:** 2017-06-29

**Authors:** Kevin C. Wang, Howard Y. Chang

**Affiliations:** 1 Program in Epithelial Biology and Department of Dermatology, Stanford University School of Medicine, Stanford, California, United States of America; 2 Veterans Affairs Healthcare System, Palo Alto, California, United States of America; 3 Center for Personal Dynamic Regulomes, Stanford University School of Medicine, Stanford, California, United States of America; Stanford University School of Medicine, UNITED STATES

Mammalian genomes are pervasively transcribed [1, 2] to produce thousands of long noncoding RNAs (lncRNAs) [3–5], transcripts that are more than 200 nucleotides in length that do not code for proteins. Although only a handful of functional lncRNAs have been well characterized to date, recent work suggests that some lncRNAs have crucial roles in the control of gene expression during both normal development and disease, through multiple mechanisms [6, 7]. As new lncRNAs are being discovered at a rapid pace, their molecular mechanisms are continuing to be enriched and diversified. For example, a few lncRNAs have been shown to affect expression of nearby genes through recruitment of protein regulatory complexes [8–10], while it is suggested that others function akin to enhancers and local regulators *in cis* [11, 12]. In fact, such local gene-regulatory mechanisms have been invoked to explain the observation that lncRNA expression is often correlated with the expression of nearby genes—the so-called “guilt by association” [13].

The *cis*-acting mechanisms of lncRNAs are largely unknown. Whether loci encoding lncRNAs function via their lncRNA transcripts or DNA elements is often unclear. An important question in the field centers around distinguishing between at least 2 possible mechanisms—either the function of many of the lncRNAs that appear to regulate genes *in cis* depends on the RNAs themselves or their effect is mediated by enhancer-like activity of underlying DNA elements in the lncRNA locus, the act of transcription, and/or splicing of lncRNAs [14, 15]. Furthermore, the molecular components regulating the expression of lncRNAs remain mostly unexplored. An understanding of the possible commonalities of the disparate underlying mechanisms may facilitate instructive and predictive models of lncRNA function.

Pradeepa et al. [16] set out to elucidate 1 mechanism of lncRNA regulation and function using the HoxA locus as their model. The Bickmore group had previously shown that positive cofactor 4 (PC4) and splicing factor 2 (SF2) interacting protein (Psip1), also known as lens epithelium-derived growth factor (LEDGF), played an important role in the regulation of Hox genes [17] and had more recently demonstrated the role of the p75 isoform of Psip1 (Psip1/p75) in recruiting the Trithorax/mixed lineage leukemia (MLL) complex to expressed Hox genes [18]. Interestingly, loss of Psip1 led not only to reduced binding of the MLL complex and loss of histone 3 lysine 4 trimethylation (H3K4me3) at the distal HoxA genes, it also resulted in complete loss of expression of the lncRNA *HoxA* transcript at the distal tip (*Hottip*), transcribed in an antisense direction away from the distal end of the 5′ HoxA cluster [10]. This suggested that Psip1 might function as a transcriptional regulator of *Hottip* expression. Following up on this possible connection, the authors showed that knockdown of Psip1/p52, the p52 isoform of Psip1, or *Hottip*, led to down-regulation of multiple 5′ HoxA genes; knockdown of p52 also strongly down-regulated *Hottip* expression. Consistent with these observations, MLL occupancy was significantly reduced across posterior HoxA genes upon knockdown of p52 or *Hottip* compared to controls.

**Fig 1:**
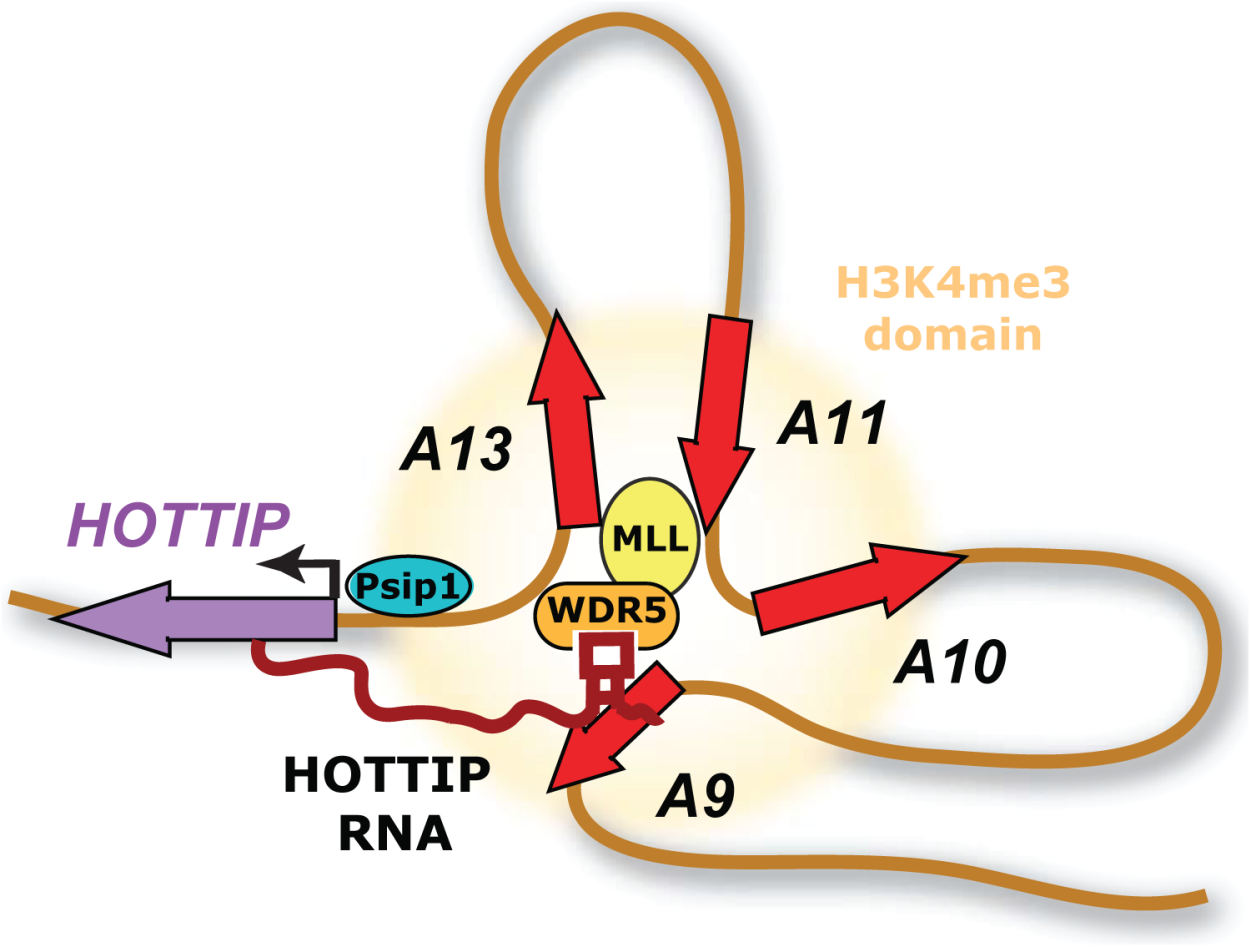
Model of positive cofactor 4 (PC4) and splicing factor 2 (SF2) interacting protein (Psip1) action. Psip1 activates the transcription of Hottip long noncoding RNA (lncRNA) in distal limb cells. Hottip lncRNA in turn stimulates the transcription of 5′ HoxA genes by enforcing H3K4me3 chromatin modification. Hottip’s selective effect on 5′ HoxA genes is due to the chromosome looping that brings 5′ HoxA genes into the vicinity of Hottip RNA emanating from the Hottip DNA locus. This mechanism transmits the spatial information in DNA looping into chemical information in chromatin modification and gene activation. Abbreviations: WDR5, WD repeat-containing protein 5; MLL, mixed lineage leukemia.

The authors next took advantage of CRISPR-Cas9 technology to create gene-body deletions of *Hottip*. Loss of *Hottip* expression led to decreased expression of posterior (*HoxA13*, *HoxA11*, and *HoxA10*) and increased expression of anterior (*HoxA2*, *HoxA6*, and *HoxA7*) HoxA genes. To find direct genomic targets of *Hottip*, chromatin isolation by RNA purification [19] (ChIRP) was performed, which demonstrated specific occupancy of *Hottip* RNA over the promoters of *HoxA13* and *HoxA11*. Importantly, ectopic activation of full-length *Hottip* via dCas9-mediated transcriptional activation showed specific induction of posterior (*HoxA13*, *HoxA11*, and *HoxA10*) but not anterior *HoxA* or *HoxD* genes. Additionally, premature termination of *Hottip* RNA by insertion of a synthetic polyadenylation cassette downstream of the *Hottip* transcription start site significantly reduced *HoxA13* and *HoxA11* mRNA levels in vitro and in vivo, firmly establishing a role for the intact *Hottip* lncRNA molecule and distinguishing its requirement from the act of transcription at the *Hottip* locus in the regulation of gene expression *in cis*. Taken together, the study provides a solid mechanism for the control of posterior *HoxA* gene transcription through activation of *Hottip* lncRNA by Psip1/p52 (Fig 1).

Several interesting and important questions remain. Psip1 binds to 5′ *HoxA* genes but somehow still requires *Hottip* RNA to activate these genes. Thus, the concept of transcription coactivator using an enhancer-like RNA to amplify or expand its reach may apply to other pairs as well. These findings also provide substantial insights into the regulation of *Hottip*, the expression of which is dysregulated in a number of human cancers. *Hottip* appears to act locally near its site of transcription, but the mechanism through which *Hottip* RNA localizes specifically to the distal *HoxA* genes *in cis* is unknown. It is possible that *Hottip* takes advantage of the existing 3-dimensional chromosomal structure at the distal HoxA loci [10] to affect the local transcription landscape. This would be an attractive mechanism given the now-appreciated highly conserved hierarchical organization of the genome [20], in which fundamental structural units serve to guide regulatory elements or, in this case, specific RNA transcripts to their cognate promoters. These interactions could provide insight into the way in which the 3-dimensional organization of the genome reflects alterations in lineage and stage-specific transcriptional programs that govern cell fate, with lncRNAs such as *Hottip* acting as well-timed molecular switches. This may represent a fundamental property of mammalian gene regulatory networks, whose mechanisms for specificity and the general applicability represent key areas for future investigation.
